# Management of Diabetes during School Hours: A Cross-Sectional Questionnaire Study in Denmark

**DOI:** 10.3390/healthcare11020251

**Published:** 2023-01-13

**Authors:** Anne Østergaard Nannsen, Kurt Kristensen, Lise Bro Johansen, Mia Kastrup Iken, Mette Madsen, Kasper Ascanius Pilgaard, Dan Grabowski, Stine Hangaard, Anders Jørgen Schou, Anette Andersen

**Affiliations:** 1 Steno Diabetes Center Aarhus (SDCA), Aarhus University Hospital, 8200 Aarhus N, Denmark; 2 Department of Pediatrics, Aarhus University Hospital, 8200 Aarhus N, Denmark; 3 Steno Diabetes Center Copenhagen (SDCC), Department of Research, Copenhagen University Hospital, 2730 Herlev, Denmark; 4 Danish Diabetes Association, 2600 Glostrup, Denmark; 5 Steno Diabetes Center North Denmark (SDCN), 9000 Aalborg, Denmark; 6 Department of Pediatrics and Adolescent Medicine, Aalborg University Hospital, 9000 Aalborg, Denmark; 7 Department of Pediatrics, Copenhagen University Hospital, 2730 Herlev, Denmark; 8 Steno Diabetes Center Odense (SDCO), 5000 Odense, Denmark; 9 Pediatric Research Unit, Odense University Hospital, 5000 Odense, Denmark

**Keywords:** diabetes management, school setting, pediatric diabetes, children with diabetes

## Abstract

Managing diabetes is complicated for many children. It often requires support from an adult during the school day. In Denmark, most children spend 30–35 h a week at school. Nevertheless, diabetes management in schools remains largely uninvestigated. This study aimed to examine the characteristics and organization of diabetes management in Danish primary schools from the personnel’s perspective. All primary schools in Denmark were invited to participate in the study (*n =* 2129), and 525 schools were included. A questionnaire was constructed and sent by email. Questionnaire data are presented in the descriptive statistics and compared with the ISPAD guidelines. According to 77.2% of respondents, school personnel had received training in diabetes management, and 78.5% of the schools had at least one person available for diabetes support every day. Respondents felt prepared to help the students with counting carbohydrates (38.9%), dosing insulin (39.1%), and helping the students during high (52.1%) or low (60.3%) blood sugar levels, insulin chock (35.2%), or during activities (36.3%). Yet, diabetes management was a challenging task. Only 61.7% had an action plan for diabetes management, 37.4% had face-to-face information meetings with the parents, and 55.1% of respondents reported having sufficient time to cooperate with the parents.

## 1. Introduction

Type 1 diabetes is one of the most frequent chronic conditions in children, with an estimated prevalence of 1.2 million cases worldwide [1]. The European region has the highest number of children and adolescents with type 1 diabetes (*n =* 295,000) and the highest numbers of incident cases in 2021 (*n =* 31,000) [1]. In Denmark, 3100 children and adolescents (0–19 years) are diagnosed with type 1 diabetes [1]. Having diabetes may complicate everyday life for the child as diabetes management requires support from an adult [2,3,4,5]. 

Most children spend many hours at school. In Denmark, children spend an average of 30–35 h a week at school [6]. This transfers the responsibility for diabetes management to the school personnel, and it may be a demanding task to help the children manage diabetes during the school day. A Swedish survey investigating the parents’ perspective of school management of diabetes found that many children did not have a contact person or an action plan in case of hypoglycemia, and the parents regularly gave less insulin in the morning due to fear of hypoglycemia during school hours [7,8].

The Convention on the Rights of the Child from 1989 is the backbone of many national laws on children’s rights around the world [9]. It states that governments should remove all obstacles for children with disabilities to make them independent and able to enjoy the best possible life in their community [9]. Therefore, Danish national law requires all schools to provide a school day on equal terms with peers for all children [10,11]. Nevertheless, Denmark has no national guidelines on what defines good diabetes care in a school setting. Diabetes management during school hours relies on the pediatric diabetes departments, schools, and parents to accommodate the individual needs of the child. 

The International Society for Pediatric and Adolescent Diabetes (ISPAD) issued the latest Clinical Practice Consensus Guidelines (ISPAD guidelines) in 2018 [12]. Whereas the guidelines of the American Diabetes Association include recommendations that are context specific to the US [13], the ISPAD guidelines are applicable worldwide [12].

It remains uninvestigated how Danish schools accommodate children with diabetes during school hours. Therefore, the aim of the study was to examine the characteristics and the organization of diabetes management in Danish primary schools as seen from the school personnel’s perspective. We used the ISPAD guidelines for diabetes management in school settings to compare the Danish level with the international recommendations. 

## 2. Materials and Methods

### 2.1. Design and Study Population

The Kids with Diabetes in School (KIDS) study is a multicenter project, which is conducted in collaboration between the Danish Diabetes Association and the five Danish Steno diabetes centers. 

A questionnaire (KIDS questionnaire) was sent to all primary schools in Denmark, including all elementary schools (0th grade, 1st–9th grade and the optional 10th grade), private primary schools (0th–9th grade), boarding schools (9th and 10th grade), and day schools (9th and 10th grade). Our survey required one respondent per school to fill in the questionnaire, and the school was instructed to hand over the questionnaire to the employee with the best knowledge on diabetes management at the given school. 

### 2.2. Data Collection 

**Figure 1:**
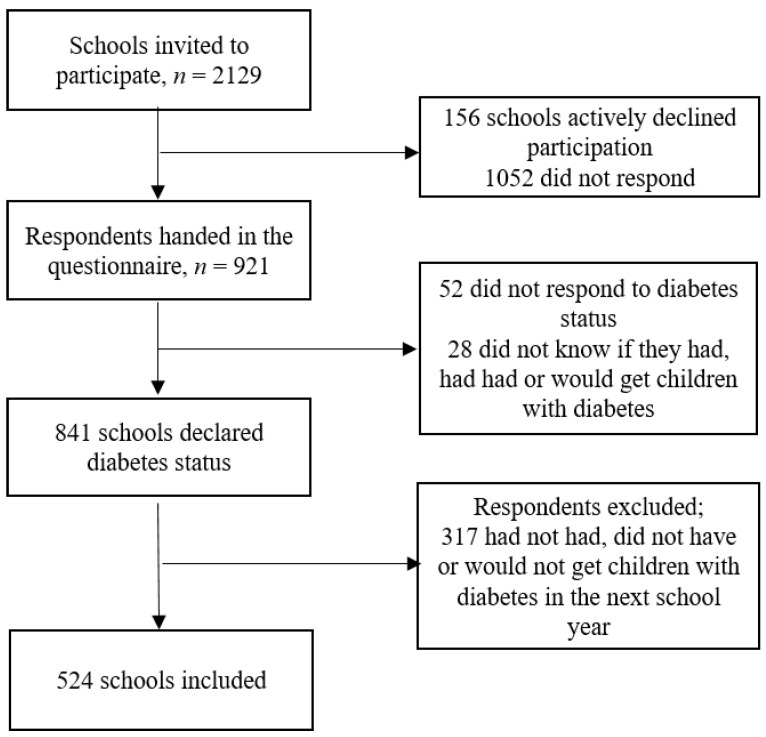
Flowchart of the inclusion into the study.

The schools were identified from the list of schools under the Danish Ministry of Children and Education. This list comprised 2129 eligible schools at the time of inclusion [14]. The questionnaire was sent to the publicly listed email address of the school. After a reminder by e-mail and then by telephone, 921 schools responded to the initial questions, 841 schools responded to the questions on diabetes status (of which 524 had children with diabetes enrolled) and were ultimately included in the study. See details in Figure 1.

### 2.3. Development of the Questionnaire

We conducted a literature search to gain insight into questionnaires on diabetes management in school settings from the personnel’s perspective. The search yielded limited results [15]. Therefore, a questionnaire was developed specifically for this study. A thorough development process preceded the final questionnaire. The purpose of the questionnaire was to explore the organization of diabetes management in Danish primary schools. We used a bottom-up approach to construct a questionnaire specifically for the Danish context based on the literature [15] and the professional expertise from multiple professions, such as medical doctors, anthropologists, and public health researchers with experience in survey studies.

The questionnaire was divided into three themes: (1) general demographic information about the school (6 items), (2) specific questions about diabetes management at the school (29 items), and (3) perceived competences and perceptions of the respondent regarding diabetes management (7 items) (Appendix A, Table A1). The questionnaire was developed and distributed in Danish and translated to English only for the purpose of this publication. 

### 2.4. Analysis

Characteristics on participating schools are presented in percent and absolute numbers (%, *n*) in Table 1. Values for less than 5 respondents are concealed and displayed as *n* ≤ 5. Data from the questionnaires were based on self-reports, except for e-mail, school name, municipality, and regional placement. First, we constructed 10 domains based on the content of the ISPAD guidelines. Second, we grouped the specific recommendations from the ISPAD guidelines under these domains. Third, we compared the questions in our questionnaire to the guidelines. The specific guideline and related questionnaire questions were then placed under a domain. This was done to ensure that the questionnaire covered the ISPAD guidelines sufficiently before we analyzed the responses from the schools. Each domain must be covered with a satisfactory response from the schools to indicate whether or not the schools meet the international standards for diabetes management in schools. Criteria for satisfactory responses from participating schools were determined a priori. For example, the school had to respond with a “yes” to give a satisfactory response to questions regarding individualized diabetes plans. We used the ISPAD guidelines to compare diabetes management in Danish primary schools with international recommendations. An overview of the ISPAD guidelines, the corresponding questions from the KIDS questionnaire, and consensus on satisfactory responses can be seen in detail in Table A1. 

The analysis of non-respondents showed that a higher proportion of non-respondents (compared to respondents) were located in the Capital Region of Denmark and Region Zealand, whereas a higher proportion of respondents (compared to non-respondents) were located in the Central Denmark Region (Table 2). Stratification on municipality [16] showed no significant differences between respondents and non-respondents (Table 2). The proportion of primary schools was higher among non-respondents (compared to respondents), and the proportion of private schools were higher among respondents (compared to non-respondents).

## 3. Results

Most participating schools were located in the Central Denmark Region (*n* = 150). Across the regional location of the schools, the majority of respondents were school principals from primary schools in areas of average wealth. Across regions, the schools were fairly evenly distributed, with <199 to 800 enrolled students (Table 1). 

### 3.1. Law and Equal Opportunity

**Figure 2:**
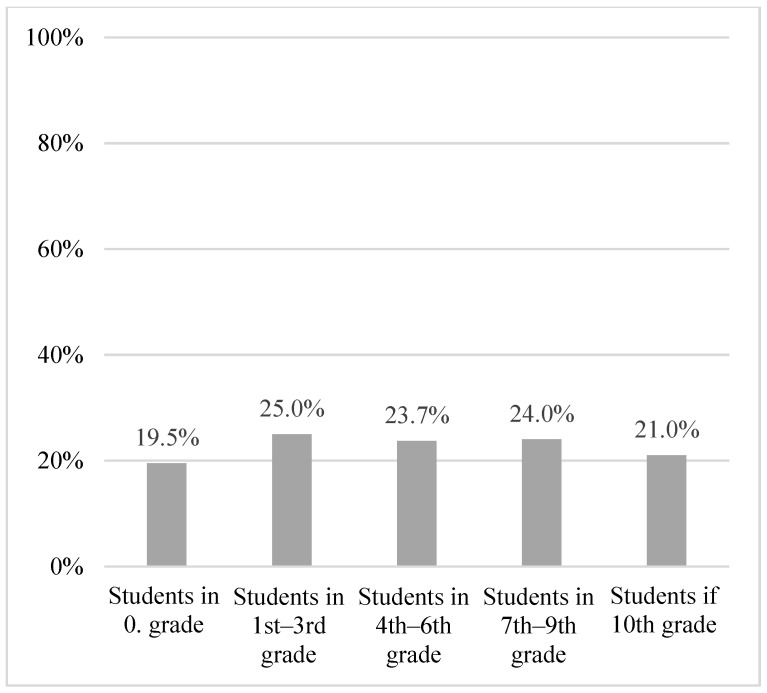
Proportion of schools allocating extra hours to students with diabetes, stratified by grade.

**Figure 3:**
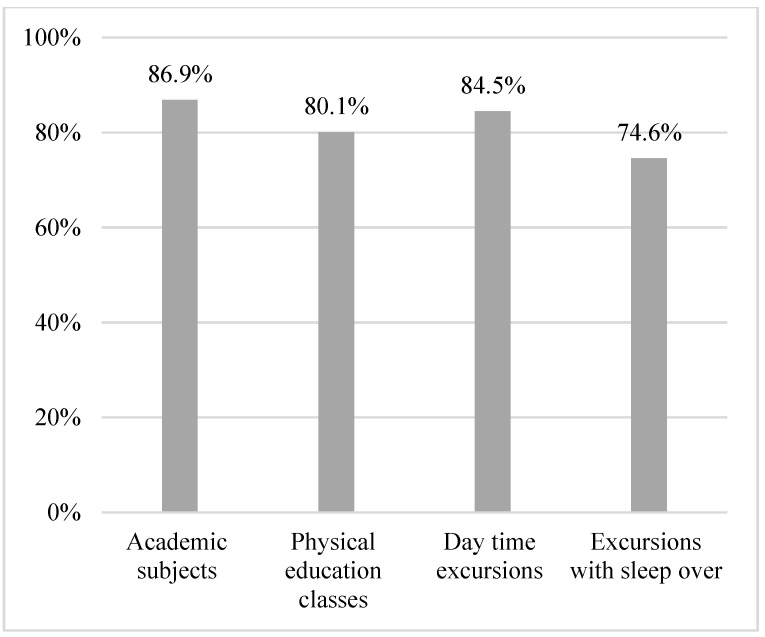
Proportion of participants feeling able to include students with diabetes during a school day.

In total, 21.7% of schools had guidelines on chronic illness in general, and 25.9% had specific guidelines on diabetes. A proportion of the schools allocated extra hours for support of students with diabetes in 0th grade (19.5%), 1st–3rd grade (25.0%), 4th–6th grade (23.7%), 7th–9th grade (24.0%), and 10th grade (21.0%). The majority of the respondents experienced the school as being able to include students with diabetes on equal terms with their peers in terms of academic subjects (86.9%), physical education (80.1%), daytime excursions (84.5%), and overnight excursions (74.6%) (Table 3, Figure 2 and Figure 3 and Table A1). 

### 3.2. Individualized Diabetes Plan

The respondents indicated that 61.7% of schools had an action plan in case of hypoglycemia, and 78.5% of schools had one or more personnel present at the school to support the students in diabetes management (Table 3 and Table A1). 

### 3.3. Education and Knowledge

**Figure 4:**
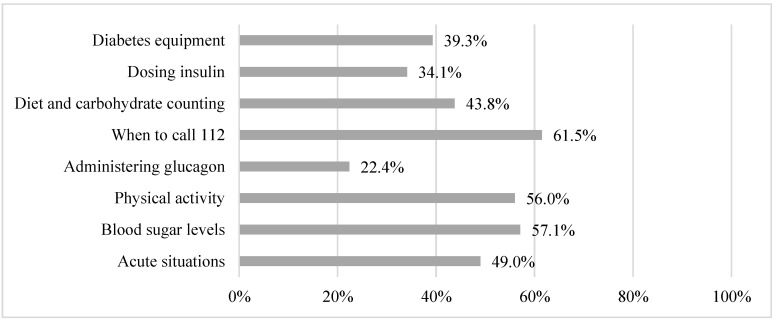
Proportion of participants with adequate knowledge on multiple subjects.

**Figure 5:**
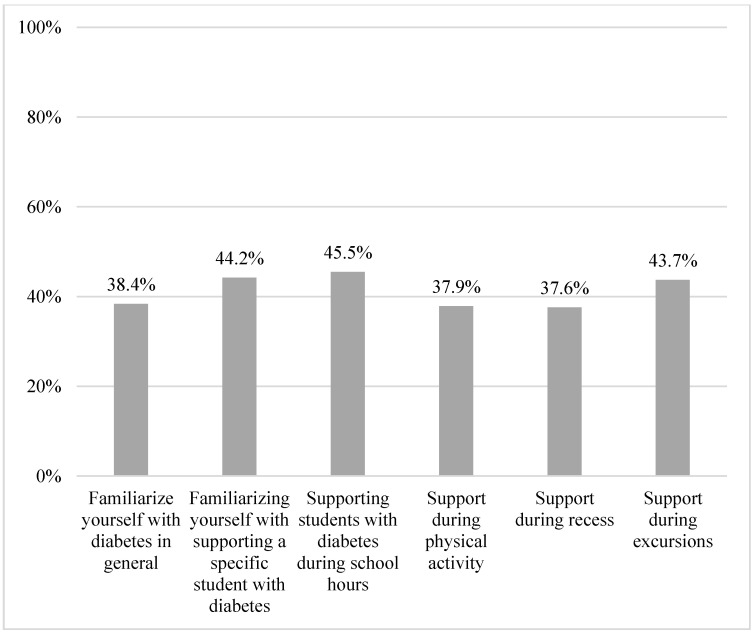
Proportion of participants with adequate time to support.

The respondents indicated that the school personnel had received diabetes specific training at 77.2% of the schools. However, the respondents reported to have inadequate knowledge on acute situations (49.0%), blood sugar levels (57.7%), impact of physical activity (56.0%), administration of glucagon (22.4%), when to call the emergency services (61.5%), and the influence of diet and carbohydrate on blood sugar levels (39.3%). In total, 54.3% of the respondents did not consider it a major challenge to have students with diabetes (Table A1). The respondents reported having adequate time to study or familiarize with diabetes in general (38.4%); to help a specific student with challenges of diabetes (44.2%); to support students with diabetes during school hours (45.5%); and to support students during physical activity (37.9%), during recess (37.6%), or during school excursions (43.7%) (Table 3, Figure 4 and Figure 5 and Table A1).

**Figure 6:**
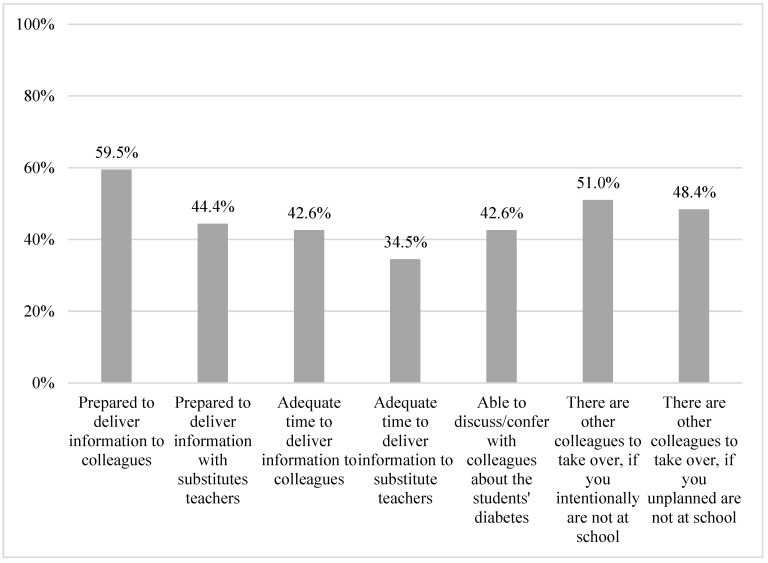
Participants’ reports on experiences with colleagues.

Regarding daily communication at school, 59.5% felt well prepared to transfer information about diabetes during the day to colleagues, and 44.4% felt well prepared to transfer information to substitute teachers. Less than half of the respondents reported to have adequate time during the day to transfer information to colleagues (42.5%) or substitute teachers (34.5%). Additionally, respondents reported on the ability to confer with colleagues about the student’s diabetes (Figure 6) (42.6%).

**Figure 7:**
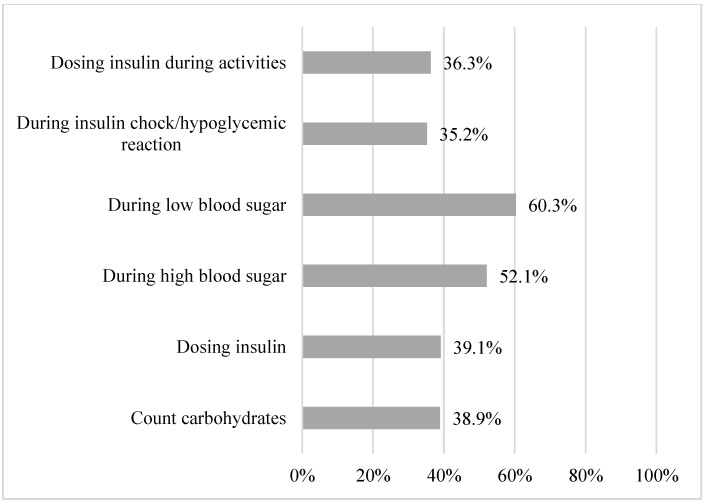
Proportion of participants prepared to help the student.

If the person with the primary responsibility of the child’s diabetes care was absent from the school, 51.0% stated having colleagues who could take on the responsibilities for diabetes care if planned in advance, and 48.4% stated having colleagues on standby who could take on the responsibilities if the absence was more acute (Table A1). The respondents stated feeling prepared to help the students with counting carbohydrates (38.9%), dosing insulin (39.1%), helping during high (52.1%) or low (60.3%) blood sugar levels, insulin chock (35.2%), or activities (36.3%) (Figure 7 and Table A1).

### 3.4. Food (Access)

In total, 72.9% of schools did not have rules regarding eating or drinking during the day (Table 3 and Table A1). Those who did have rules allowed the students with diabetes to eat or drink as needed if they consulted an adult first (data not shown). 

### 3.5. Physical Activity

The majority of schools reported having no diabetes-related limitations in school activities, including physical activity (95.0%). In some situations, for example excursions, 69.7% of schools were able to bring in extra personnel (Table 3 and Table A1). 

### 3.6. Self-Management 

**Figure 8:**
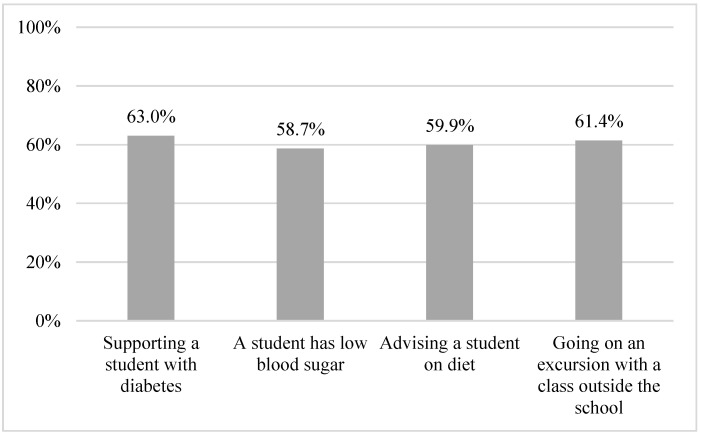
Proportion of participants not feeling unsafe.

At 85.7% of the schools, the respondents estimated that students with diabetes did not lose considerable time during class due to diabetes management, and 84.9% of respondents found that these children did not lose time during recess. The majority of schools made it possible for students to have blood sugar levels measured during the day (97.4%). Most schools had no rules regarding use of restroom (96.2%) or use of mobile phones at all times (53.0%). The respondents reported on the extent to which they felt unsafe in performing tasks related to diabetes management, (1 or 2 on a five-point scale) for supporting a student with diabetes (63.0%), supporting a student with low blood sugar level (58.7%), supervising a student on diet (59.9%), or going on school excursions with a student with diabetes (61.4%) (Table 3, Figure 8 and Table A1). 

### 3.7. School/Home Cooperation 

**Figure 9:**
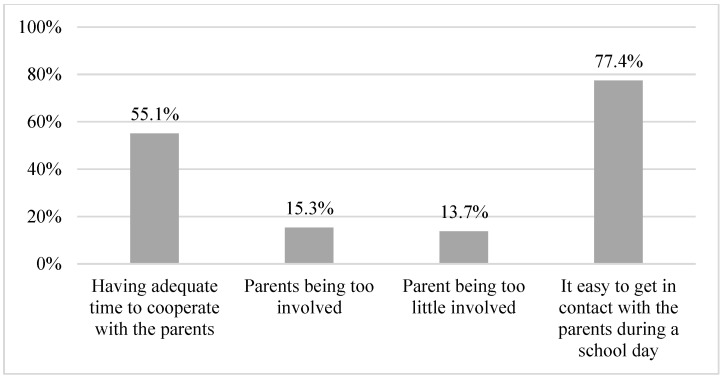
Proportion of schools with parent involvement .

In total, 37.4% of the schools had information delivered face-to-face to the parents. The respondents reported that 81.4% of parents gave relevant information on diabetes management and that 84.6% gave the necessary supplies for diabetes management. The parents were perceived to be neither too much involved (15.3%) nor too little involved (13.7%). In addition, 55.1% of the respondents reported having adequate time to cooperate with the parents, and 77.4% found it easy to contact parents during the school day (Table 3, Figure 9 and Table A1).

## 4. Discussion

A large proportion of the Danish primary schools were found to have organized their diabetes management in accordance with the ISPAD guidelines. The results indicate that the majority of the schools are capable of including children with diabetes on equal terms with their peers as stipulated in the Danish national law. The majority of schools had at least one person available to support diabetes management during the day, but the results also suggest several areas for improvement of diabetes management in Danish schools.

Only a quarter of the schools have adopted specific guidelines for diabetes management, and only around 60% had an action plan in case of hypoglycemia, which can be life- threating if not treated promptly. At many of the schools, the personnel had received diabetes specific training. Nonetheless, they still felt inadequate and unprepared in several key aspects of diabetes management, such as managing blood sugar levels, diet, and acute situations. These results are supported by a German study from 2020 investigating teachers’ perceptions of diabetes management, which found low confidence among teachers when doing daily diabetes management and low institutional support, such as written instructions and diabetes specific policies [17]. A Swedish study from 2017 investigating school management in diabetes from a parental perspective found similar proportions of schools with a written action plan [18].

Most respondents saw no limitations for children with diabetes in school activities, including physical activity. Still, in half of schools, the respondents reported feeling underprepared in supporting the children during physical activity. Less than 25% of the schools allocate extra hours to support students with diabetes, which indicates that many schools do not allocate extra hours for support. This might place a huge burden on the teachers who support the child as they must do so along with their regular teaching hours. It may also place greater demands on the parents to support the child during the day. Kingod et al. reported that some parents receive several phone calls during the day from the school to get help in diabetes management, and the advice is thus very dependent on the parents receiving diabetes management training [4]. Haslund Thomsen et al. found that lower levels of confidence and knowledge of diabetes management among school personnel greatly affect how much parents have to be a part of the school day [19].

Approximately 80% of schools have one or more personnel present every day. This means that 20% of the schools may lack experienced support for children with diabetes. Additionally, only about half of the schools explicitly stated that diabetes management is not considered a challenge, but a considerable proportion of personnel reported to feel unprepared in performing specific diabetes management tasks. This may place heavy demands on the personnel, both professionally and personally. Our data showed that the respondents in 40% of schools felt less safe when supporting students with diabetes. A Swedish study from 2017 also found limited personnel to support the child with diabetes management during the school day [18]. Lack of training and little knowledge of diabetes management was also reported in a Spanish study, which found that 43.2% of survey respondents did not have enough knowledge about type 1 diabetes although they had previously had or currently had children or adolescents with type 1 diabetes [20]. Our results are similar to a German study, which also found limited communication between teachers and parents about medical needs and a lack of written policies and plans on diabetes management [17].

These results indicate that a discrepancy exists between what the schools convey to provide for the children and the more personal experiences of navigating diabetes management among the school personnel. The results also suggest a lack of structural support in diabetes management, such as appropriate guidelines, action plans, face-to-face meeting with parents, and insufficient time to cooperate with the parents.

### 4.1. Strengths and Limitations 

To the best of our knowledge, this study is the first to explore school personnel’s perspectives on and perceptions of diabetes management in a nationwide Danish school setting. The study is also the first to provide insight into the organization and daily management of diabetes in schools in Denmark.

Knowledge on the subject is scarce. We were inspired to use the ISPAD guidelines as it was important to use a questionnaire encompassing a wide range of many aspects of diabetes management in a school setting. Using the ISPAD guidelines ensured that all key themes were considered. The questionnaire in this study included questions related to all aspects of the ISPAD guidelines, except for psychosocial environment. We wanted to explore treatment aspects of diabetes management, knowledge and feelings of safety in diabetes management for school personnel, but we were not able to fairly evaluate the efforts of the school to accommodate the psychosocial aspects of school life for children with diabetes. The student’s psychosocial environment is important and should be explored in future studies. Currently, there are no official guidelines on this topic in Denmark or in the Nordic countries. Therefore, we deemed the internationally applicable ISPAD guidelines suitable for comparison for two reasons. First, they present a set of broad recommendations to schools worldwide. Second, they provide caregivers (at home and in school) with standards to guide diabetes management [12,13]. The guidelines encompass many key aspects of diabetes management, but they may not address the Danish school system in all aspects. For example, primary schools in Denmark are not obligated to provide lunch for the children. Therefore, when we evaluate food access in primary schools according to the ISPAD guidelines, we are evaluating whether the food is accessible in general (as provided by the parents), but not whether the school provides food. Schools are only obligated to provide lunch in boarding schools, where the children live at the premises. Further, our survey included multiple questions on inclusion of children with diabetes during a school day, rather than just asking about inclusion in physical education classes as in the ISPAD guidelines. This provided us with a broader insight into all aspects of a school day. 

The present study was further strengthened by the pilot-testing of the questionnaire among several groups of potential respondents prior to the actual survey. A large proportion of the ISPAD guidelines are contained in the Danish national law, such as the legal obligation to provide a school day on equal terms (see Table A1, section “Law and equal opportunity”) [12]. This requires schools to support the child with diabetes management on a daily basis, e.g., measurement of blood sugar level. The ISPAD guidelines state that personnel should be legally authorized to support the child. The Danish law states that schools are legally obligated to support the child. This discrepancy between the ISPAD guidelines and Danish national law makes it difficult to fulfil this part of the ISPAD guidelines. Therefore, tailoring the ISPAD guidelines to a Danish context would be appropriate.

Our study also has some limitations. As it was not possible to identify where children with diabetes go to school, it was not possible to target the survey at only schools with children with diabetes enrolled. Therefore, we were able to examine only non-respondents for demographic data, but we could not calculate response rates for schools with children with diabetes. There was a risk of misclassifying the respondents based on their reports on students with diabetes enrolled, as it is currently not possible to verify these reports. The self-reported data in this study hold a risk of underreporting or overreporting among respondents. The respondents were school personnel with knowledge on children with diabetes. In many cases (*n* = 265), the respondent was the school principal and not the child’s closest teacher. Thus, we cannot rule out that this could have influenced the answers and thereby have underestimated or overestimated certain aspects of diabetes management.

We invited all primary schools in Denmark to participate in the study. In total, 524 schools were eligible for the study based on the enrolment of children med diabetes. Several factors could have influenced the selection into the study. The data collection was conducted during the COVID-19 pandemic, where restrictions burdened the daily management in schools, and new teaching situations could have affected the school’s willingness to participate. Still, primary schools tend to be busy in general, and similar participation rates have been found in other Danish studies [21,22]. Our analysis of non-respondents showed there were more non-respondents in the regions close to the capitol. The reasons for this are unclear. One explanation could be that the eastern part of the country was battling higher numbers of COVID-19 cases for an extended period during the data collection for this study and may not have prioritized participation, or it could be a management decision not to participate. More private primary schools participated than public primary schools. We hypothesize that this might be related to variations in available resources during school days between public and private primary schools. Several schools might only have one child with diabetes and may not consider participation as relevant. Lastly, the person receiving the questionnaire initially may not have known whether or not the school had children with diabetes, but this person still had to decide whether or not to participate. Therefore, the study population might underrepresent the true population, which holds a risk of underestimating or overestimating the results. 

Future research using this data should include hypotheses testing, for example concerning how the characteristics of diabetes management and the perceptions of the school personnel are associated.

### 4.2. Perspectives to Future Work in the Field 

Only a third of the respondents reported having enough time to prepare for and get acquainted with diabetes management. Some schools had appointed a contact person (other than the teacher) for the child. Schools with experience in diabetes management could also serve as advisors for schools without such experience. The situation could be less vulnerable if the school had a small team of teachers and other personnel who could help the child if the assigned contact person was absent (as there would be someone else to cover the diabetes management for a short period of time). Several respondents felt inadequate and unprepared when supporting a student with diabetes. It is essential that school personnel feel capable of caring for the students. One option to strengthen this capability could be setting up different types of learning options to help the personnel, such as e-learning modules, revisiting the training program for diabetes management, apps for managing diabetes, and tele-support from diabetes clinics and other schools with similar experience. Another option could be to organize visits from diabetes clinics at the schools as part of the training of teachers (instead of making the teachers go to the clinics) as this is likely to enhance accessibility and participation, including repeat sessions and brush-up courses. Working with the personnel’s qualifications and feelings of safety in managing daily diabetes tasks could alleviate the burden on both school personnel and parents. A third option could be to organize network meetings with various stakeholders that are relevant for children with diabetes to explore new ideas for diabetes management and to keep up with new developments in diabetes management. National guidelines need to be developed in Denmark to accommodate the difficulties experienced by school personnel and to ensure that the necessary resources are available for the personnel to feel capable of providing diabetes management support. National guidelines are important because they consider the context and the circumstances that are specific for Denmark.

Future studies should also focus on validating the KIDS questionnaire, which has not yet been validated. This is likely because no similar questionnaires seem to exist in Danish to validate against. Validating the questionnaire would make us able to verify the results and conduct follow-up studies with repeated measures.

## 5. Conclusions

In this study, diabetes management was found to be a challenging task for the staff in primary schools, as some personnel felt inadequate and unprepared in several key aspects of diabetes management. Our results indicate a lack of structural support in diabetes management. The respondents pointed to the absence of guidelines and action plans, few face-to-face meetings with parents, and little time to cooperate with the parents.

## Figures and Tables

**Table 1:** Characteristics of participating schools stratified on regional location, *n =* 524.

	Capital Region of Denmark	Central Denmark Region	North Denmark Region	Region Zealand	Region of Southern Denmark
Participants, % (*n*)	19% (101)	29% (150)	13% (66)	12% (65)	27% (142)
Institution, % (*n*)					
*Primary school (public)*	63% (64)	67% (100)	65% (43)	58% (38)	49% (69)
*Primary school (private)*	34% (34)	19% (29)	24% (16)	35% (23)	35% (49)
*Boarding school*	*n* ≤ 5	13% (19)	11% (7)	*n* ≤ 5	15% (21)
*Day school*	*n* ≤ 5	*n* ≤ 5	0% (0)	0% (0)	*n* ≤ 5
Number of students per school, % (*n*)					
*<199 students*	15% (15)	31% (46)	45% (28)	31% (20)	34% (48)
*200–399 students*	19% (19)	23% (35)	26% (17)	31% (20)	31% (44)
*400–599 students*	21% (21)	21% (21)	18% (27)	25% (16)	19% (27)
*600–799 students*	24% (24)	19% (29)	11% (7)	12% (8)	11% (16)
*800–999 students*	13% (13)	7% (10)	*n* ≤ 5	*n* ≤ 5	*n* ≤ 5
*>1000 students*	9% (9)	*n* ≤ 5	0% (0)	0% (0)	*n* ≤ 5
Respondents’ occupation, % (*n*)					
*Administrative personnel*	14% (14)	15% (23)	*n* ≤ 5	15% (10)	12% (17)
Teacher	14% (14)	19% (29)	17% (11)	14% (9)	27% (38)
*Social worker*	7% (7)	6% (9)	9% (6)	8% (5)	6% (8)
*Principal*	53% (53)	49% (74)	67% (44)	45% (29)	46% (65)
*Other manager than principal*	11% (11)	5% (7)	*n* ≤ 5	8% (5)	7% (10)
*Other, non-manager*	*n* ≤ 5	5% (8)	0% (0)	11% (7)	*n* ≤ 5
Area (wealth), % (*n*)					
*Not so wealthy*	13% (13)	16% (23)	35% (23)	24% (15)	19% (26)
*Very wealthy*	8% (8)	3% (5)	0% (0)	0% (0)	*n* ≤ 5
*Some wealth*	33% (33)	17% (25)	11% (7)	19% (12)	18% (25)
*Not at all wealthy*	*n* ≤ 5	4% (6)	9% (6)	*n* ≤ 5	5% (7)
*Average*	44% (44)	60% (89)	45% (30)	51% (32)	57% (80)

**Table 2:** Analysis of non-respondents stratified on region, municipality, and institution, *n* = 2129.

	Non-Respondents	Respondents
*n* (%)	1052 (66.8%)	524 (33.2%)
Region, *n* (%)		
*Capital Region of Denmark*	275 (26.1%)	101 (19.3%)
*Central Denmark Region*	184 (17.5%)	150 (28.6%)
*North Denmark Region*	103 (9.8%)	66 (12.6%)
*Region Zealand*	213 (20.2%)	65 (12.4%)
*Region of Southern Denmark*	277 (26.3%)	142 (27.1%)
Municipalities, *n* (%)		
Capital municipalities	216 (20.5%)	99 (18.9%)
*Metropolitan Municipalities*	51 (4.8%)	46 (8.8%)
*Provincial municipalities*	238 (22.6%)	95 (18.1%)
*Commuter Municipalities*	227 (21.6%)	112 (21.4%)
*Rural Municipalities*	320 (30.4%)	172 (32.8%)
Institution, *n* (%)		
*Boarding school, live in*	95 (9.0%)	53 (10.1%)
Primary schools	673 (64.0%)	314 (59.9%)
*Private primary school*	225 (21.4%)	151 (28.8%)
*Boarding schools*	59 (5.6%)	6 (1.1%)

**Table 3:** Overview of single-item questions and subsequent proportion of satisfactory responses.

	Proportion of Satisfactory Response
Does the school have a guideline/policy for handling chronic illness among students?	21.7%
Does the school have a guideline/policy for handling diabetes among students?	25.9%
Does the school have an action plan, in case a students with diabetes experiences low blood sugar/insulin chock (hypoglycemic reaction)?	61.7%
Does the school have one or more persons able to help students with diabetes administer insulin/adjust insulin pump if necessary?	78.5%
Did teachers and other personnel receive teaching regarding supporting students with diabetes?	77.2%
What do you think is the biggest challenge for the school, in regards to management and support to students with diabetes?	54.3%
Are there any rules for where and when students with diabetes are allowed to eat/drink?	72.9%
Are there activities at school where students with diabetes are limited/restricted?	95.3%
It is possible for the school to have extra personnel on hand in some situations, for example excursions?	69.7%
Are the students able to measure blood sugar levels at the school?	97.4%
Are there any rules for where and when students with diabetes are allowed to use their phones?	53.0%
Are there any rules for when and where students with diabetes are allowed to use the restroom?	96.2%
Are students able to manage their diabetes, for example eat/drink, measure blood sugar without losing considerable time during class?	85.7%
Are students able to manage their diabetes, for example eat/drink, measure blood sugar without losing considerable time during recess?	84.9%
How is information from the parents delivered?	37.4%

## Data Availability

Data can be made available upon request.

## Appendix A

**Table A1:** Overview of the ISPAD guidelines, KIDS questionnaire and satisfactory responses, (*n* = 524).

Domain: Law and Equal Opportunity				
ISPAD Guidelines	KIDS Questionnaire Items	Questionnaire Response Categories	Criteria for Satisfactory Response	Proportion of Schools with Satisfactory Response
Irrespective of age and ability, all students with diabetes at school must receive the support, encouragement, and supervision of school personnel Optimal management of diabetes at school is a prerequisite for optimal school performance, including learning, and for the avoidance of diabetes-related complications Maintaining normo-glycemia during school hours is important and day-to-day glycemic targets should not differ from any other setting Diabetes is classified by “common law” as a disability and legal frameworks exist in many nations to ensure the child has equal opportunity to participate in all aspects of school life Many children with diabetes worldwide do not have ready access to insulin, diabetes supplies, or education. They should be given the same opportunity as other children to obtain an education School exams or other assessment situations are associated with stress and increased risk of acute transient episodes of hypoglycemia or hyperglycemia that can affect performance Administration, or careful supervision, of insulin administration requires school personnel to be legally authorized with informed parental consent Schools should make “reasonable adjustments” to facilitate prescribed medical care to allow for children with type 1 diabetes (T1D) to participate in education on the same basis as their peers. Schools have a non-delegable duty of care to their students, and school personnel should take reasonable care to protect them from harm that is reasonably foreseeable.	Does the school have a guideline/policy for handling chronic illness among students?	□ Yes □ No	Yes	21.7%
	Does the school have a guideline/policy for handling diabetes among students?	□ Yes □ No	Yes	25.9%
	Does the school allocate extra hours to students with diabetes? State approximate amount of time for each age group: 1. Students in 0th grade 2. Students in 1st–3rd grade 3. Students in 4th–6th grade 4. Students in 7th–9th grade 5. Students in 10th grade	Text box	>1 h	1. 19.5% 2. 25% 3. 23.7% 4. 24% 5. 21%
	On a scale from 1 to 5 (5 being the highest), to what extent do you experience being able to include students with diabetes in a school day on equal terms with students without diabetes during…. 1. Academic subjects 2. Physical education classes 3. Day time excursions 4. Overnight excursions	Likert scale: 1–5	For all: 4–5	1. 86.9% 2. 80.1% 3. 84.5% 4. 74.6%
Domain: Individualized diabetes plan				
ISPAD guidelines	KIDS questionnaire items	Questionnaire response categories	Criteria for satisfactory response	Proportion of schools with satisfactory response
All young people with diabetes at school should have an individualized diabetes management plan (DMP) in place which must be developed and agreed with parents in advance. The DMP should be reviewed and amended as and when necessary, according to the needs of the young person with diabetes, and/or at least annually. The type of insulin regimen used at school should be tailored to the needs, ability, and wishes of the child/family and should not be dictated by the school resources. Whether children can self-manage certain aspects of their diabetes and/or self-administer insulin is not necessarily age dependent and can only be determined by the parent and health care team. Specific arrangements may need to be put in place (including access to BG testing equipment; hypoglycemia first-aid pack) for exams	Does the school have an action plan, in case a students with diabetes experiences low blood sugar/insulin chock (hypoglycemic reaction)?	□ Yes □ No □ Don’t know	Yes	61.7%
	Does the school have one or more persons able to help students with diabetes administer insulin/adjust insulin pump if necessary?	□ Yes, there is one person at the school □ Yes, there are more than one person at the school □ No □ Don’t know	Yes	78.5%
Domain: Education and knowledge				
ISPAD guidelines	KIDS questionnaire items	Questionnaire response categories	Criteria for satisfactory response	Proportion of schools with satisfactory response
Schools are responsible for adequately training their personnel about diabetes, but the content of the training is the responsibility of the health care team and parent. School personnel should be aware of the signs/symptoms of hypoglycemia, and a “first-aid hypoglycemia” management pack should be available at all times. Clear instructions for managing hypoglycemia should be provided. Blood glucose (BG) monitoring is central to achieving optimal glycemic control at school and must be familiar to school personnel. “Reasonable adjustments” include school personnel support with insulin administration, as well as understanding and knowledge of diabetes technologies (including continuous glucose monitoring [CGM] devices and insulin pump settings). School personnel should be able to manage appropriately the effects of low and high BG levels according to parent and health care team instructions.	Did teachers and other personnel receive teaching regarding supporting students with diabetes?	□ Yes, all personnel have received teaching □ Yes, personnel, involved with students with diabetes have received teaching □ No □ Don’t know □ Feel free to elaborate:_____	Yes	77.2%
	What do you think is the biggest challenge for the school, in regards to management and support to students with diabetes?	□ It is not considered a challenge to have students with diabetes at the school □ Lack of teaching/instruction in management of diabetes among students □ Teachers cannot/will not take on the responsibility for students with diabetes □ There is not a teacher or pedagogue with knowledge of diabetes present at school every day □ The school nurse is not present every day □ The school has a lot of students with chronical illnesses/health challenges □ The school needs guidelines in this area □ Other:________	It is not considered a challenge to have students with diabetes at the school	54.3%
	On a scale from 1–5 (5 being the highest), to what extent do you feel like you have adequate knowledge about…. 1. Acute situations 2. Blood sugar levels 3. Physical activity 4. Administering glucagon 5. When to call 112 6. Diet and carbohydrate counting 7. Dosing insulin 8. The students diabetes equipment	Likert scale: 1–5	For all: 4–5	1. 49.0% 2. 57.1% 3. 56.0% 4. 22.4% 5. 61.5% 6. 43.8% 7. 34.1% 8. 39.3%
	On a scale from 1–5 (5 being the highest), to what extent do you feel you have adequate time to… 1. Familiarize yourself with diabetes in general 2. Familiarizing yourself with supporting a specific student with diabetes 3. Supporting students with diabetes during school hours 4. Support during physical activity 5. Support during recess 6. Support during excursions	Likert scale: 1–5	For all: 4–5	1. 38.4% 2. 44.2% 3. 45.5% 4. 37.9% 5. 37.6% 6. 43.7%
	On a scale from 1–5 (5 being the highest), to what extent do you experience… 1. Being well prepared to deliver information to colleagues 2. Being well prepared to deliver information to substitutes teachers 3. You have adequate time to deliver information to colleagues 4. You have adequate time to deliver information to substitute teachers 5. You are able to discuss/confer with colleagues about the students’ diabetes 6. There are other colleagues to take over, if you intentionally are not at school 7. There are other colleagues to take over, if you unplanned are not at school	Likert scale: 1–5	For all: 4–5	1. 59.5% 2. 44.4% 3. 42.6% 4. 34.5% 5. 42.6% 6. 51.0% 7. 48.4%
	On a scale from 1–5 (5 being the highest), to what extent do you feel you are prepared to… 1. Help the student with counting carbohydrates 2. Help the student with dosing insulin 3. Help the student during high blood sugar 4. Help the student during low blood sugar 5. Help the student during insulin chock/hypoglycemic reaction 6. Help the student dosing insulin during activities	Likert scale: 1–5	For all 4–5	1. 38.9% 2. 39.1% 3. 52.1% 4. 60.3% 5. 35.2% 6. 36.3%
Domain: Food (Access)				
ISPAD guidelines	KIDS questionnaire items	Questionnaire response categories	Criteria for satisfactory response	Proportion of schools with satisfactory response
Access to food in schools is an integral part of enabling children to grow normally and balance their insulin and food intake Use of food pictures may help school personnel assess food servings and their estimated carbohydrate content	Are there any rules for where and when students with diabetes are allowed to eat/drink?	□ Yes, they are only allowed to eat/drink in consultation with personnel □ No, we do not have any rules for □ Don’t know	No, we do not have any rules	72.9%
Domain: Psychical activity				
ISPAD guidelines	KIDS questionnaire items	Responsecategories in the questionnaire	Criteria for satisfactory response	Proportion of schools with satisfactory response
All young people with T1D should be given the same opportunities as their peers to participate safely in all sports and physical activity	Are there activities at school where students with diabetes are limited/restricted?	□ No, there are no limitation/restrictions □ Yes, daytime excursions with the school □ Yes, overnight excursions □ Yes, physical education (PE) class □ Yes, swimming lessons □ Yes, bigger sports events □ Yes, at after school programs for older students □ Yes, at after school programs for younger students □ Other activities:_____	No, there are no limitations/restrictions	95.3%
	It is possible for the school to have extra personnel on hand in some situations, for example excursions?	□ Yes □ No Feel free to elaborate:_______	Yes	69.7%
Domain: Self-management				
ISPAD guidelines	KIDS questionnaire items	Questionnaire response categories	Criteria for satisfactory response	Proportion of schools with satisfactory response
Young people with diabetes must be allowed to monitor their BG levels, administer insulin, and to treat low/high BG values at any time during the school day, with adult supervision if needed	Are the students able to measure blood sugar levels at the school?	□ Yes, at all places at school □ No, the student is not able to measure blood sugar at school □ Yes, in the classroom □ Yes, in the office area □ Yes, at the school nurse office □ Other places:_______	Yes	97.4%
	Are there any rules for where and when students with diabetes are allowed to use their phones?	□ No, they are allowed to use their phones in class and recess □ Yes, some rules □ Feel free to elaborate on any rules:____	No, they are allowed to use their phones in class and recess	53.0%
	Are there any rules for when and where students with diabetes are allowed to use the restroom?	□ No □ Don’t know □ Yes, these rules apply:____	No	96.2%
	Are students able to manage their diabetes, for example eat/drink, measure blood sugar without losing considerable time; 1.During class? 2. During recess?	□ Yes □ No □ Don’t know	Yes	1. 85.7% 2. 84.9%
	On a scale from 1–5 (5 being the highest), to what extent do you feel unsafe if… 1. You have to support a student with diabetes 2. A student has low blood sugar 3. You have to advise a student on diet 4. You have to go on an excursion with a class outside the school, where there is a student with diabetes	Likert scale: 1–5	For all: 1–2	1. 63.0% 2. 58.7% 3. 59.9% 4. 61.4%
Domain: School/home cooperation				
ISPAD guidelines	KIDS questionnaire items	Questionnaire response categories	Criteria for satisfactory response	Proportion of schools with satisfactory response
With a mutually supportive, collaborative approach between parents and the child’s health care team and schools, and with advancements in communication technology, for example, providing sensor glucose data in real time to parents, there is a real opportunity for a truly cooperative approach. Parents cannot be expected to “fill the gap” of school resources and attend to their child’s medical management during the school day. Successful diabetes management at school heavily depends on effective communication and problem-solving with the family and schools should clarify expectations and coordinate communication	How is information from the parents delivered?	□ In a meeting between parents and school □ As written materials □ In AULA or other online platforms □ Don’t know □ Anything else:_____	In a meeting between parents and school	37.4%
	Do parents of students with diabetes hand out; 1. Relevant information about management of diabetes? 2. The necessary supplies (glucagon, strips for blood glucose measurements, snacks, juice/dextrose?	□ Yes □ No □ Some parents do not □ Don’t know	Yes	1. 81.4% 2. 84.6%
	On a scale from 1–5 (5 being the highest), to what extent do you experience … 1. Having adequate time to cooperate with the parents 2. Parents being too involved 3. Parent being too little involved 4. It easy to get in contact with the parents during a school day	Likert scale: 1–5	For all: 4–5	1. 55.1% 2. 15.3% 3. 13.7% 4. 77.4%
Domain: Psychosocial environment				
ISPAD guidelines	KIDS questionnaire items	Questionnaire response categories	Criteria for satisfactory response	Proportion of schools with satisfactory response
Peer relations, local social stigma, racial and religious perspectives can be a burden to patients and families with T1D. Young people with diabetes have a significantly increased risk of being exposed to issues of discrimination, which may impact on self-esteem and cause feelings of stigmatization. Schools provide a unique opportunity to identify and treat psychological problems in young people with diabetes and close liaison between school personnel and health care professionals is recommended. Some studies report higher rates of psychological problems such as depression and eating disorders in young people with diabetes.				
